# Answering the Cell Stress Call: Satellite Non-Coding Transcription as a Response Mechanism

**DOI:** 10.3390/biom14010124

**Published:** 2024-01-17

**Authors:** Marisa Fonseca-Carvalho, Gabriela Veríssimo, Mariana Lopes, Daniela Ferreira, Sandra Louzada, Raquel Chaves

**Affiliations:** 1 CytoGenomics Lab, Department of Genetics and Biotechnology (DGB), University of Trás-os-Montes and Alto Douro (UTAD), 5000-801 Vila Real, Portugal; marisa-carvalho16@hotmail.com (M.F.-C.); gabriela.verissimo96@gmail.com (G.V.); lopesfmariana@gmail.com (M.L.); dpferreira@utad.pt (D.F.); slouzada@utad.pt (S.L.); 2 BioISI—Biosystems & Integrative Sciences Institute, Faculty of Sciences, University of Lisboa, 1749-016 Lisbon, Portugal

**Keywords:** cellular stress, satellite DNA, satellite non-coding RNAs, stress-response mechanisms

## Abstract

Organisms are often subjected to conditions that promote cellular stress. Cell responses to stress include the activation of pathways to defend against and recover from the stress, or the initiation of programmed cell death to eliminate the damaged cells. One of the processes that can be triggered under stress is the transcription and variation in the number of copies of satellite DNA sequences (satDNA), which are involved in response mechanisms. Satellite DNAs are highly repetitive tandem sequences, mainly located in the centromeric and pericentromeric regions of eukaryotic chromosomes, where they form the constitutive heterochromatin. Satellite non-coding RNAs (satncRNAs) are important regulators of cell processes, and their deregulation has been associated with disease. Also, these transcripts have been associated with stress-response mechanisms in varied eukaryotic species. This review intends to explore the role of satncRNAs when cells are subjected to adverse conditions. Studying satDNA transcription under various stress conditions and deepening our understanding of where and how these sequences are involved could be a key factor in uncovering important facts about the functions of these sequences.

## 1. Introduction

Cells can be subject to numerous internal and external factors that can trigger signaling mechanisms, namely environmental conditions (such as radiation or temperature), and responses to chemical or pathogenic agents. These conditions cause stress to the cells and promote a cellular response which is dependent on the type of stress, and also the type of cell, tissue and organism. The stress response and the consequent activation of cellular pathways have been related to mechanisms that encompass the deregulation of a series of genes [1,2,3]. Studies demonstrate that non-coding RNAs (ncRNAs), including those originating from satellite DNA sequences, are also important players in various mechanisms of cellular response to stress conditions [4].

Satellite DNAs (satDNAs) are highly repetitive sequences present in the eukaryotic genomes. These sequences were initially identified as bands with distinct densities concerning the rest of the genome [5,6] and were later shown to be organized in tandem arrays accounting for a significant amount of the total DNA content of some genomes [7]. These sequences are the main component of constitutive heterochromatin and are located primarily in the centromeric and pericentromeric regions of eukaryotic chromosomes [8,9,10]. However, they have been also found in euchromatin in shorter arrays dispersed in the genome [10,11].

The transcription of satDNAs into satellite non-coding RNAs (satncRNAs) has gathered increasing interest due to their association with important functions in the organization and regulation of the genome of several organisms: vertebrate [4,10,12,13,14] and invertebrate [15,16]. A growing number of studies report that satncRNAs are important regulators of cell processes and their deregulation has been associated with disease, namely the tumor process [16,17,18,19]. In particular, these transcripts integrate a significant fraction of differentially expressed sequences in response to various stress stimuli and their stability seems to be adjusted according to their regulatory functions, mechanisms of action and the physiological state of the cell [3].

This review explores the response mechanisms to different types of cellular stress (such as heat shock, oxidative, osmotic, DNA damage response, and emotional stress), where satDNAs transcripts, as well as variation of their number of copies, take part. We aim to discuss in what way cell stress promotes alterations of satDNA transcription levels and number of copies and elucidate the mechanisms that are triggered. This work intends to highlight the significant role of satellite DNA sequences in the stress response, evidenced by different examples in varied eukaryotic species.

## 2. Heat Shock Response Mechanisms as a Way to Counterpart Thermal and Oxidative Stress

Eukaryotic cells developed a highly conserved protective response, the heat shock response (HSR), that allows them to cope with stressful conditions, such as exposure to high temperatures or the presence of oxidants, that can cause protein misfolding and denaturation [20]. The HSR is regulated by a family of transcription factors called heat shock factors (HSFs) [21]. In the absence of stress, HSFs are typically present in an inactive form in the cytoplasm of cells. Upon exposure to heat or other stressors, heat shock transcription factor 1 (HSF1) becomes activated and translocates to the nucleus, where it binds to heat shock response elements (HSEs) in the promoter regions of heat shock genes [1,22,23,24]. These genes are rapidly upregulated in response to stress, resulting in the production of heat shock proteins (HSPs) [23,25]. HSPs are divided into different families based on their molecular weights; Hsp70, Hsp90, and Hsp60 are the best characterized [25,26]. These proteins act as molecular chaperones in cells—they work together in a network to help fold newly synthesized polypeptides, refold unstable proteins, assemble protein complexes, break up protein aggregates, and break down misfolded proteins [1,27]. Besides their chaperone functions, they also play vital roles in regulating cell signaling, cell cycle, and programmed cell death [25]. The next subsections highlight how the transcription of satDNAs relates to the HSR, essentially focusing on the upregulation of satDNAs (promoting gene expression alterations), and on the interaction between upregulated satDNA transcripts and HSR-related proteins.

### 2.1. Satellite DNA Transcription as a Shared Response to Thermal Stress among Different Species

**Figure 1:**
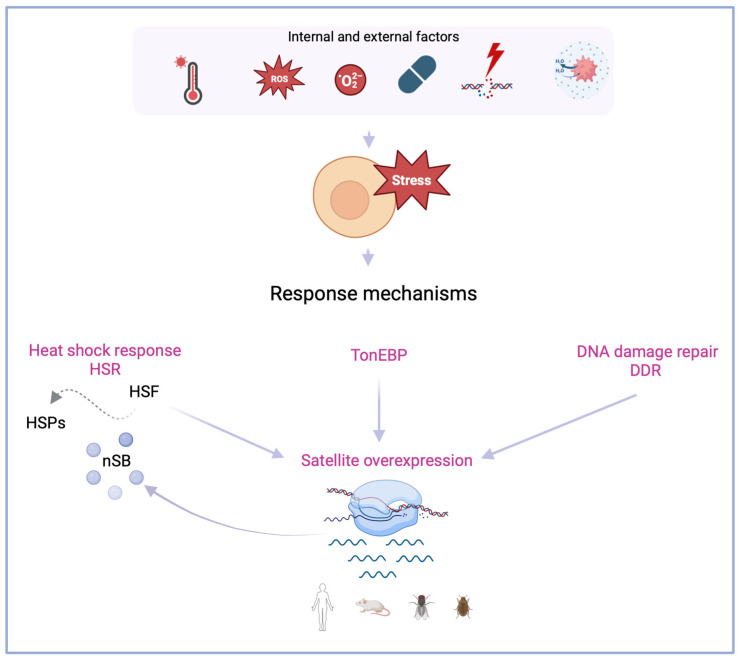
Response mechanisms when cells are subjected to internal and external factors that promote stress. Evidence shows that satellite overexpression (in various organisms, human, mouse, fly and beetle) is associated with mechanisms that act when cells respond to stress factors. The heat shock response (HSR) is regulated by heat shock factors (HSFs), and transcription factors of heat shock proteins (HSPs). Upon heat shock, nuclear stress bodies (nSBs) are formed in transcribing human satellite 3 (Hsat3) *loci*. When cells are subjected to hyperosmotic stress, the transcription factor tone enhancer (TonEBP) promotes Hsat3 transcription. Cellular exposure to physical-chemical or environmental agents can promote genome instability and the activation of DNA damage repair (DDR), which also leads to satellite overexpression. Created with BioRender.com.

The exposure to thermal stress triggers the heat shock response mechanism, leading to the cellular activation of a molecular network upon the possibility of significant damage to proteins, nucleic acids, and membrane lipids [28]. When cells are subjected to high temperatures—heat shock stress—the normal functioning of cellular processes is disrupted to deal with the induced cellular damage. In the event of failure to mitigate the consequences, programmed cell death is the last resource [3]. Studies across several animal species have shown that heat shock stress can lead to changes in the organization and expression of satDNA sequences [22]. The mentioned changes were found to be deeply intertwined. Heat shock stress leads to the increased expression of satDNAs. This increased transcription seems to depend on the localization of the original sequence. Heterochromatic tandemly repeated satellites are particularly sensitive to heat shock, which appears to be a shared characteristic of upregulated satDNAs between different species. The organization effects of satDNA overexpression can be an outcome of changing the heterochromatic landscape. The epigenetic remodeling of heterochromatin post-heat shock can promote recombination between genomic and extrachromosomal satDNA arrays, which leads to repeat exchanges between heterochromatic and euchromatic sites [29,30], ultimately responding differently to stress conditions. SatDNA upregulation has been analyzed in several species: mammals, such as *Homo Sapiens* [17,31,32,33] and *Mus musculus* [34,35]; but also insects, such as *Tribolium castaneum* [16,36,37] and *Drosophila melanogaster* [38,39,40] (Figure 1).

**Figure 2:**
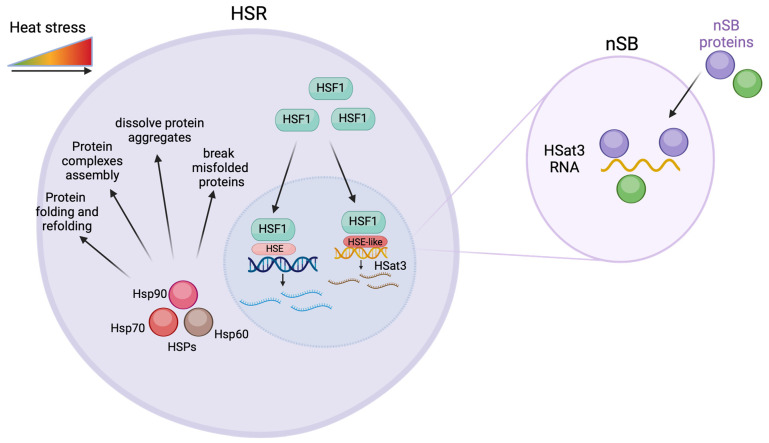
Overexpression of HSat3 transcripts and the heat shock response (HSR). When cells are under heat stress, Heat shock factor (HSF) is translocated to the nuclei where it binds to heat shock elements (HSE) leading to the expression of heat shock proteins. The HSF also promotes the expression of HSat3 through an HSE-like factor. The HSat3 transcripts interact with nuclear stress bodies proteins in the nuclear stress bodies (nSB). Created with BioRender.com.

The involvement of satncRNAs, namely human satellite 3 (Hsat3) transcripts, in the human heat stress response mechanism is the best-characterized relation between these types of transcripts and cellular stress conditions. Heat shock (HS) has already been shown to induce the transcription of HSat3 repeat arrays located at the pericentromeric heterochromatin of specific human chromosomes (Table 1) [41]. HSat3 transcription has been described in chromosome 9 (*locus* 9q12) [31,35] and, more recently, in the Y chromosome (*locus* Yq12) [42]. These chromosomal locations are coincident with large arrays of HSat3 [43,44,45]. The transcription of HSat3 pericentromeric heterochromatin relates to several proteins: transcription factors, such as Heat Shock Factor 1 (HSF1) [46] and CREB-binding protein (CREBBP) [17]; serine/arginine-rich splicing factors 1 and 9 (SRSF1 and SRSF9) [47] and various RNA-binding proteins [48]. During heat shock, the activation of HSF1, and possible recognition of HSat3 HSE-like elements promote HSat3 transcription (Figure 2) [49]. Chromosomes 9 and Y are both characterized as primary targets of HSF1 [21].

The nuclear accumulation of HSat3 transcripts has been associated with the formation in *cis* of specific structures known as nuclear stress bodies (nSBs) (Figure 2) [12,17,42]. Subsequent to HSat3 transcription, nSBs colocalize with HSF1, CREBBP, RNA polymerase II, and splicing factors [49,50]. HSat3 ncRNA-rich nSBs were found to promote the transcriptional repression necessary for HSR (Figure 2). During heat shock, the transcriptional upregulation of HSPs is accompanied by global transcriptional repression. The loss of HSat3 ncRNA alleviates this transcriptional repression. More specifically, knocking down HSat3 transcripts demonstrated that the recruitment of CREBBP and SRSF1 to nSBs is dependent on this satncRNA [48]. The same study also revealed that the overexpression of HSat3 transcripts in the absence of heat shock still triggers nSBs formation and gene repression [48]. Furthermore, the identification of HSat3 ncRNA-associated RNA-binding proteins suggests that HSat3 transcripts serve as a platform to sequester dephosphorylated serine/arginine splicing factors (SRSFs). This event can be quickly followed by their release from nSBs, after rephosphorylation by protein kinase 1 (CLK1)—also recruited by HSat3 transcripts. This promotes a rapid adaptation of gene expression during and after exposure to heat stress [51,52].

**Table 1:** Overexpression of satellite DNA sequences from different species under cellular stress.

Stress	Condition	Satellite	Species	References
Heat stress	High temperature	HSat3	*Homo sapiens*	[12,41]
		HSat2	*Homo sapiens*	[53,54]
		αSat	*Homo sapiens*	[32]
		γ-satellite	*Mus musculus*	[55]
		TCAST1	*Tribolium castaneum*	[56]
		hsrω	*Drosophila melanogaster*	[38,39,40]
Oxidative Stress	H_2_O_2_	HSat3	*Homo sapiens*	[12]
	PDX5 knockdown	HSat3	*Homo sapiens*	[57]
		αSat	*Homo sapiens*	[57]
Osmotic Stress	Sorbitol	HSat3	*Homo sapiens*	[12]
DNA damage response	UV-C	HSat3	*Homo sapiens*	[12]
	5-FU, Cisplatin	HSat3	*Homo sapiens*	[33]
	Zeocin, Etoposide	HSat2	*Homo sapiens*	[58]
	Cytostatic drugs	γ-satellite	*Mus musculus*	[55]
	Zeocin, Etoposide	MiSat MaSat	*Mus musculus*	[34]

Human pericentromeric GC-rich satellite DNA 2 (HSat2) sequence, was also demonstrated to be overexpressed in heat shock stressed cells (Table 1) [53,54]. The overexpression of Hsat2 transcripts can reflect global changes in heterochromatin silencing [53], namely causing the overexpression of other satDNA sequences. Increased HSat2 transcription in response to heat shock pathway hyperactivation during tumorigenesis could lead to the targeted demethylation of the HSat3 1q12 *locus* [54]. Additionally, human centromeric α satellite DNA (αSat) has also been the subject of studies that revealed a substantial increase in its transcription levels when cells are subjected to thermal stress (Table 1). This increase is followed by an enrichment of the histone 3 lysine 9 trimethylation (H3K9me3) histone mark in αSat sequences dispersed throughout the genome, including in regions of euchromatin. This enrichment in H3K9me3 was proposed to be a mechanism of epigenetic remodeling to reorganize heterochromatin after heat shock [32]. Another study demonstrated that the application of heat shock followed by the upregulation of satDNA transcription leads to the accumulation of death domain associated protein (Daxx; involved in the maintenance of nuclear homeostasis) on (peri)centromeric heterochromatin, to protect the heterochromatic epigenetic state [59].

SatDNA overexpression under heat shock cellular stress was also described in rodents. The highly conserved mouse *Cassini* ncRNA (murine γ-satellite) was revealed to be upregulated in acute lymphoblastic leukemia (ALL) cells under conditions of heat shock, even though we still have no indication if these transcripts are strategically involved in the stress response or have adverse consequences (Table 1) [55].

Some insect species have also shown increased transcription of satDNAs when cells undergo heat stress. The major satellite of *Tribolium castaneum* (TCAST1) is demethylated and overexpressed upon heat shock (Table 1) [56]. This is part of a mechanism to restore silent histone modifications (H3K9me2/3) at heterochromatic satellite repeats during heat shock recovery [16,36,37], as mentioned above for αSat transcripts [32]. However, when comparing transcripts from the major satellite TCAST1 and minor satellite TCAST2, the latter is not induced by heat shock [37], possibly due to its preferred location in euchromatin [30]. *Drosophila melanogaster* also showed an increased transcription of hsrω satDNA during heat shock (Table 1) [38,39,40]. Hsrω transcripts seem to accumulate at the *locus* of origin during HS, as what happens with HSat3 transcripts. Hsrω and HSat3 ncRNAs associate with similar RNA processing factors [17,31,60] and, therefore, may be functionally comparable [38]. More precisely, the recovery of heat shock in *Drosophila melanogaster* is dependent on hsrω transcripts for the relocation of several proteins to an original status: heterogeneous nuclear ribonucleoproteins (hnRNPs), heterochromatin protein 1 (HP1), and RNA polymerase II [40]. These target proteins are also known to interact with HSat3 transcripts in humans [17,47,61].

Heat stress is one of the environmental stressors that have been extensively studied in relation to its impact on satDNA transcription. Regardless of the species or the specific satDNA sequences being considered, there is a consistent trend of increased transcription of satDNA sequences under conditions of heat stress. As already proposed for humans and mice, satDNAs might share inducibility [55], which suggests a potentially shared functional role. Recent research indicates that these transcripts might not be mere byproducts of heat stress-induced transcription, but rather active players in cellular stress responses [22].

### 2.2. Satellite DNA Transcription in the Response to Oxidative Stress

Oxidative stress is characterized by an imbalance between reactive oxygen species (ROS) production and antioxidant defense [18,62], which can cause damage to cellular components such as proteins, lipids, and DNA, potentially leading to cellular dysfunction and disease [63,64]. ROS are highly reactive molecules that are produced naturally as sub-products of cellular metabolism and under stressful conditions, also serving as signaling molecules, and influencing a range of cellular processes [65]. Eukaryotic cells are particularly vulnerable to oxidative stress because they contain organelles such as mitochondria and peroxisomes that produce ROS as part of their normal function [63,66,67]. ROS can damage mitochondrial components, creating a feedback loop wherein mitochondrial dysfunction results in further ROS production [68]. The cellular response to oxidative stress involves intricate mechanisms designed to counteract the potential damage inflicted by excessive ROS by means of nonenzymatic and enzymatic mechanisms [63,69]. Persistent oxidative stress can prompt cells to embark on distinct paths. It may trigger apoptosis, a programmed cell death mechanism that eliminates cells carrying irreparable damage, thereby averting further harm [63]. Alternatively, oxidative stress can drive cellular senescence, a state of irreversible growth arrest that prevents the proliferation of cells that may pose a risk due to accumulated DNA damage [70].

Human satellite 3 RNA has been shown to be induced by multiple stress agents, such as oxidative stress when cells were treated with H_2_O_2_ (Table 1) [12]. This treatment has been shown to moderately increase the expression of HSat3 as well as trigger the formation of nSBs in a small number of cells [12]. Upon oxidative stress, the levels of antioxidants modulate the activation of the heat shock factor and consequently, the production of heat shock proteins [71]. The activation of HSF1 via ROS results from an indirect pathway involving the activation of transcription factors like nuclear factor erythroid 2 related factor 2 (Nrf2) [18,72]. Nrf2 orchestrates the transcription of genes encoding antioxidant enzymes and detoxification proteins, bolstering the cellular defense against oxidative stress-induced harm [72,73].

In line with this, in humans, an increase in the centromeric αSat and HSat3 transcripts was detected in a study that encompasses the knockdown of peroxiredoxin-5 (PRDX5), an antioxidant protein that neutralizes ROS in human lung cancer cells [57]. PRDX5 is a peroxiredoxin involved in antioxidant defense and redox signaling [74]. In light of the mentioned findings, αSat and HSat3, are indicated to have their transcription regulated by the PRDX5 gene, which crucially safeguards the genome from oxidative damage (Table 1) [57].

The transcriptional activity of major satellite (MaSat) after the induction of retinoic acid was analyzed in rat embryonic cells. An increase in MaSat transcripts was observed after retinoic acid induction and cell differentiation accompanied by changes in this satDNA [75]. Retinoic acid exhibits antioxidant properties and activates pathways that enhance antioxidant defense, enabling cells to counteract oxidative stress and promote DNA repair while maintaining redox balance [76]. Thus, retinoic acid’s antioxidant effects and activation of protective pathways will be able to help cells mitigate oxidative stress, potentially leading to an increase in satDNA transcripts, and further contributing to cellular adaptation. Furthermore, this study suggests that RNA helicase p68 (a multifunctional protein involved in nuclear processes) interacts with MaSat, possibly acting as a transcription regulator [75]. Thus, the RNA helicase p68 role can facilitate the regulation of specific sequences, including MaSat, by interacting with factors influenced by retinoic acid.

## 3. The Relation between Osmotic Stress and Satellite DNA Transcription

Osmotic stress occurs when there is an excessive amount of salt in the surrounding environment, which promotes changes in external osmolarity resulting in the disruption of ions and water balance within the cell [77]. When eukaryotic cells are under osmotic stress, ROS production is triggered as a consequence of altered metabolic processes, particularly at the mitochondria electron transport chain [78]. Notably, ROS function as secondary messengers, instigating the activation of crucial signaling pathways [79]. For instance, osmotic stress-associated ROS are known to influence mitogen-activated protein kinases (MAPK) and calcium signaling, both important in the cell’s adaptive response to osmotic challenges [80]. As osmotic stress activates ROS production, cells mobilize their antioxidant defense mechanisms to avert potential damage [79]. Antioxidant enzymes like superoxide dismutase, catalase, and glutathione peroxidase are deployed to neutralize ROS and reinstate redox equilibrium [73]. Moreover, ROS are not merely passive participants; they actively shape gene expression patterns by engaging transcription factors [81]. These factors, such as nuclear factor-κB (NF-κB), activator protein-1 (AP-1), and protein 53 (P53) are pivotal in initiating the expression of genes that contribute to the cell’s stress response and adaptation [73,79].

It has been described that hyperosmotic stress also induces the transcriptional activation of HSat3, involving a transcription factor tonicity enhancer binding protein, the transcription factor tone enhancer (TonEBP) (Table 1) [12]. The TonEBP transcripts also accumulate at the 9q12 *locus* (location of HSat3) [35], just as with HSF during heat-shock. In addition, TonEBP is also known for the activation of T-cell nuclear factor 5 (NFAT5), described to regulate gene expression in response to osmotic stress and to be vital in kidney function and protection against high levels of salinity [82,83,84].

## 4. The Pathways of DNA Damage Response (DDR) Encompass Satellite DNA Transcription

Cellular exposure to the molecules that are produced by physical-chemical or environmental agents, such as ultraviolet radiation, ionizing radiation, and chemotherapy drugs, can promote genome instability. This cellular outcome is related to the development of several diseases and to avoid them, cells developed DNA repair pathways [85]. Despite the lack of research regarding the function of satncRNAs in these DNA damage response (DDR) mechanisms, some works suggest that they can be key players in protecting the cells against these factors [86,87].

Ultraviolet radiation (UV-A, UV-B, UV-C) can cause double-stranded DNA breaks (DSBs) [88,89], which results in cell cycle arrest through the recruitment of ATM (ataxia-telangiectasia mutated) protein kinase that mobilizes one of the most extensive signaling networks as an attempt to repair them, and when it is not possible, the apoptosis pathway is triggered by P53 protein [90,91]. Although studies are still very limited, there is already evidence that cellular exposure to this type of radiation can influence the transcription of satDNA sequences. Valgardsdottir and colleagues (2008) described the increased transcription of HSat3 after UV-C radiation treatment (Table 1). They proposed that UV-C stress also triggers the heat-shock response and the formation of structures similar to nSBs [12]. Some authors suggest that these nSBs may protect the large heterochromatic block at 9q12 from chromosomal rearrangements induced by stress (Table 1) [17]. Another study that focused on the functional characterization of human HSat2 in DDR showed that the UV-C stress induction in arising retinal pigment epithelia (ARPE-19) cells does not result in a significant alteration of its transcription levels, possibly as a consequence of the type of DNA damage and its cellular outcome (Table 1) [58].

DDR mechanisms can be also triggered by cytotoxic agents, i.e., the association of satncRNAs aberrant expression with chromosomal instability (CIN) and DDR [92,93,94]. In 2018, Ichida and colleagues demonstrated that the overexpression of αSat led to CIN and copy number changes at specific chromosomes [95]. Despite the growing number of studies that highlight the relation between drug response and satncRNA levels, the mechanisms are still poorly understood [94]. Human cells treated with zeocin (an antibiotic that mimics the effect of ionizing radiation on cells) or etoposide (an anticancer agent that causes DSBs and prevents their repair by Topoisomerase II) showed an increased expression of HSat2 RNA (Table 1) of about 500 and 700 times, respectively. This accumulation of transcripts is regulated by DDR mechanisms and it is not dependent on P53 pathways [58]. It has been also demonstrated that HSat3 transcripts can promote resistance to etoposide and that the epigenetic modification of the HSat3 *locus* and its expression can be used to predict the response to this treatment. The same authors suggest that HSat3 transcripts are capable of recruiting topoisomerase II alpha (TOP2A) to the nSBs in response to stress. This will result in resistance to etoposide treatment because it prevents the formation of the etoposide-TOP2A complex, and consequently, the decrease in DNA damage. However, it is possible to restore etoposide sensitivity when HSat3 expression is reduced [96]. Another study supports these data and relates HSat3 overexpression to resistance to chemotherapeutic drugs such as staurosporine, fluorouracil (5-FU), and cisplatin (interferes with the cell cycle and target caspase-specific cell death) (Table 1) [33]. HSat3 knockdown restores the P53 function, promoting cell death, and sensitizing cells to these agents. Also, P53 regulates the levels of HSat3 ncRNAs to induce cell death. These authors suggest that HSat3 is an important regulator of human cancers, facilitating cancer progression by a different pathway from the heat stress pathway [33]. Some works on mouse satellite RNAs also showed the importance of satncRNA in DDR. First, the *Cassini* satellite (from the mouse γ-satellite family) was shown to be upregulated in acute lymphoblastic leukemia cells when they are treated with cytostatic drugs [55]. More recently, a study demonstrated the relation between the overexpression of the mouse major satellite RNA (MaSat) and DDR features, namely abnormal segregation (involving micronuclei formation and anaphase bridging) and increased levels of the DNA damage marker γ phosphorylated form of the histone H2AX (γH2AX). The authors also showed a relation between overexpression of MaSat and sensitivity to camptothecin (CPT) (topoisomerase I inhibitor) via CIN induction [94]. After etoposide or zeomicin treatment in murine cells, it was also observed the increased expression of MaSat and minor satellite repeats (MiSat), being this last more evident (Table 1) (Figure 1). However, this increase in MiSat is dependent on P53 presence, suggesting that a stabilized form of P53 can bind to non-canonical sites in MiSat repeats, activating its transcription in the DDR signaling pathway [34].

## 5. Beyond Satellite Transcription: Satellite DNA Copy Number Variation in Response to Emotional Stress during Aging and Disease

Although most work focuses on the analysis of satDNA transcription, the number of copies of these satDNAs can also be associated with several biological processes and diseases. Recently, Ershova and colleagues (2019) associated the HSat3 copy number variation with aging. The sub-fraction of HSat3 located at the 1q12 region (characterized for being a rather unstable region of the human genome) was studied in a group of healthy individuals with ages ranging from two to ninety-one years, separated into different age groups. HSat3 was quantified using a specific probe by means of a non-radioactive quantitative hybridization method, and it was observed that in young people the number of copies of HSat3 was much lower than in the group of elderly people aged between 77 and 91 years [97]. The authors associated the increase in the number of copies with emotional stress caused by environmental changes (social conditions and radiation exposure) to which the studied population was subject. The population born between 1912 and 1925, experienced the First World War, Russian Revolution and Russian Civil War (living in unfavorable social conditions), and according to the data obtained, it was the population group that had a greater number of copies. The latter finding is similar to the results obtained in the analysis of individuals who were born between 1975 and 2000 and exposed to high levels of radiation (Chernobyl incident), and to the stressful conditions caused by the former Soviet Union, and to individuals from the group 1926 to 1975, that worked with ionizing radiation sources. Realizing that variations in the number of copies of satDNA sequences are related to environmental factors is an interesting path that can help in the deeper knowledge of different pathologies and psychiatric illnesses, such as schizophrenia.

Schizophrenia is a serious, disabling and chronic psychiatric illness characterized essentially by auditory, visual and olfactory hallucinations [98]. It was recently demonstrated that patients with schizophrenia have a lower number of copies of HSat3 when compared to healthy individuals (quantification by non-radioactive hybridization) [99]. HSat3 content in patients diagnosed with schizophrenia varies across different brain areas [100]. Moreover, it has been reported that patients submitted to antipsychotic therapy show an increase or decrease in the number of copies of HSat3, depending if they have initially a low or high HSat3 content, respectively [100]. It is known that the number of copies of HSat3 is influenced by both endogenous (variable number of copies between individuals in the same population) and exogenous (environmental stresses such as ionizing radiation and oxidative stress conditions) [97,101]. This may indicate two possible scenarios, either the low amount of HSat3 is a general feature of schizophrenia patients, or these patients’ genomes react to chronic oxidative stress (caused by this disease) by reducing the number of HSat3 copies [100]. This realization opens a new path to investigate the role of satellite DNAs in psychiatric diseases, accounting that they may share common features. In this sense, more of this type of illness has to be studied regarding these repetitive sequences state in patients versus healthy individuals, not only in terms of the number of copies but also in terms of its transcription levels and possible regulatory functions. We believe that, similarly to what happens with other types of diseases, the study of satncRNAs in this specific area will unravel some of the mechanisms in which they are involved.

## 6. Concluding Remarks

Evidence has proven the relevance of satDNA sequences in varied biological processes. Along this review, we presented several examples that validate the important role of these sequences in cellular responses that are triggered when the cell is under stress conditions. Most of the sequences described here show an alteration of their transcriptional profile when cells face adverse stressful conditions, and the majority of them seem to share common features, such as their pericentromeric location in the genome. A future avenue to explore is the possibility that the chromatin environment of satellite sequences influences their activity under stress. Heterochromatic pericentromeric sequences can perhaps share transcriptional inducibility. This feature could be explained by what happens in heat shock with heterochromatic TCAST and preferably euchromatic TCAST2, the former transcriptionally active in heat shock, and the latter not altered [36].

The heat shock response is the most explored pathway, being promoted by different types of stresses, like temperature and oxidative damage, that result in the overexpression of satDNAs. Sequences like the human HSat3 and the insect’s TCAST satellites, are so far the best studied when it comes to their participation in stress response. Hence, to explore the shared inducibility hypothesis, transcriptional studies should be extended to other satellite sequences in a wide range of species. It would also be important to study whether, in response to stress, satellite transcripts act as a protective cellular mechanism, promoting rapid transcriptional changes. This knowledge could be achieved by multiple satellite silencing experiments, and the subsequent assessment of the concerted molecular pathways of stress response (such as HSR, acting in various stress types). Likewise, the study of several satellite sequences could disclose if their role is active or bystander-like.

In addition, the changes in the satDNA number of copies and its association with emotional stress or schizophrenia give us a new perspective that can be further explored. Future research can address whether the copy number alterations directly reflect transcriptional changes in response to cellular stress.

The stress pathways described in this review share the following trait: satDNA expression changes in response to stress, which triggers gene expression alterations. This opens up the possibility of addressing satellite ncRNAs from an epigenetic perspective and searching for possible interactors of gene expression regulation. Although there is a significant number of reports on satellite DNA transcription in several species, not much is known regarding its regulation. All the above highlights the need to perform further studies involving satellite DNA sequences to understand the precise mechanisms underlying the relationship between satDNA and stress response. Besides, there is also the need to develop new experimental tools that can address the functional aspects of these repetitive sequences. Such information will allow a deeper understanding of satellite DNA’s physiological role under regular and stress conditions.

## List of Abbreviations

5-FU	Fluorouracil
ALL	Acute lymphoblastic leukemia
AP-1	Activator protein-1
ARPE-19	Arising retinal pigment epithelia
ATM	Ataxia-telangiectasia mutated
CIN	Chromosomal instability
CLK1	Protein kinase 1
CPT	Camptothecin
CREBBP	CREB-binding protein
Daxx	Death domain associated protein
DDR	DNA damage repair
DSBs	Double-stranded DNA breaks
H3K9me2/3	Histone 3 lysine 9 trimethylation
hnRNPs	Heterogeneous nuclear ribonucleoproteins
HP1	Heterochromatin protein 1
HS	Heat shock
HSat2	Human Satellite 2
HSat3	Human Satellite 3
HSE	Heat shock elements
HSF1	Heat shock transcription factor 1
HSFs	Heat shock factors
HSPs	Heat shock proteins
HSR	Heat shock response
Hsrω	Heat-shock RNA omega
MAPK	Mitogen-activated protein kinases
MaSat	Major satellite
MiSat	Minor satellite
ncRNAs	Non-coding RNAs
NF-κB	Nuclear factor-κB
NFAT5	Nuclear factor of activated T-cells 5
Nrf2	Nuclear factor erythroid 2 related factor 2
nSBs	Nuclear stress bodies
P53	Protein 53
PRDX5	Peroxiredoxin-5
ROS	Reactive oxygen species
satDNA	Satellite DNA
satncRNAs	Satellite non-coding RNAs
SRSF1	Serine/arginine-rich splicing factors 1
SRSF9	Serine/arginine-rich splicing factors 9
SRSFs	Serine/arginine-rich splicing factors
TCAST1	*Tribolium castaneum* the major satellite
TonEBP	Transcription factor tone enhancer
TOP2A	Topoisomerase II alpha
αSat	Alpha satellite DNA
γH2AX	γ phosphorylated form of the histone H2AX
